# Dynamic Pathway Selectivity of TAS2R5 toward or Away from β‑Arrestin or G Protein from Biased Agonists

**DOI:** 10.1021/acs.biochem.6c00174

**Published:** 2026-05-16

**Authors:** Donghwa Kim, Hannah R. Strzelinski, Camille M. Longabardi, Stephen B. Liggett

**Affiliations:** † Department of Internal Medicine, 33697 University of South Florida Morsani College of Medicine, Tampa, Florida 33602, United States; ‡ Center for Personalized Medicine and Genomics, University of South Florida Morsani College of Medicine, Tampa, Florida 33602, United States; § Department of Molecular Pharmacology and Physiology, University of South Florida Morsani College of Medicine, Tampa, Florida 33602, United States

## Abstract

Bitter taste receptors (TAS2Rs) are GPCRs functionally expressed in extraoral organs including the lung, heart, brain, and gastrointestinal system and are candidate targets for novel drugs. It is unclear whether TAS2R5 can be stabilized by structurally distinct agonists resulting in pathway selectivity (biasing) to achieve optimal outcomes. We screened TAS2R5 agonists for signaling toward or away from β-arrestin or G protein ([Ca^2+^]_i_). Agonists T5-1 (1,10-phenanthroline) and T5-6 (4,7-dimethyl-1,10-phenanthroline) coupled equally to G protein but showed marked differences in homologous desensitization (∼21% and ∼91%, respectively). This desensitization was due to dissimilar degrees of β-arrestin engagement compared to the reference agonist T5-7: T5-6 evoked up to ∼86% enhancement of β-arrestin1 or β-arrestin2 recruitment, while T5-1 was biased in the opposite direction, up to ∼78% less, compared to T5-7. T5-1 elicited little receptor internalization compared to T5-6 consistent with the β-arrestin findings. For the initial response, T5-1 and T5-6 represent one-pathway differences (decreased or increased β-arrestin) leading to bias in different directions, while G protein signaling was equivalent. However, under desensitizing conditions T5-6 was decoupled from G protein representing two-pathway biasing in opposing directions for T5-6 (increased β-arrestin and decreased G protein), imposing extreme biasing of β-arrestin over G protein. Our finding that agonists can affect β-arrestin biasing in either direction suggests that TAS2R5 is sufficiently pliable for specific signals to be engineered depending on the desired therapeutic outcome. The dynamic nature of the directionally contrasting changes of the two pathways over time accentuated bias and is due to the interaction of the two signals.

## Graphical abstract

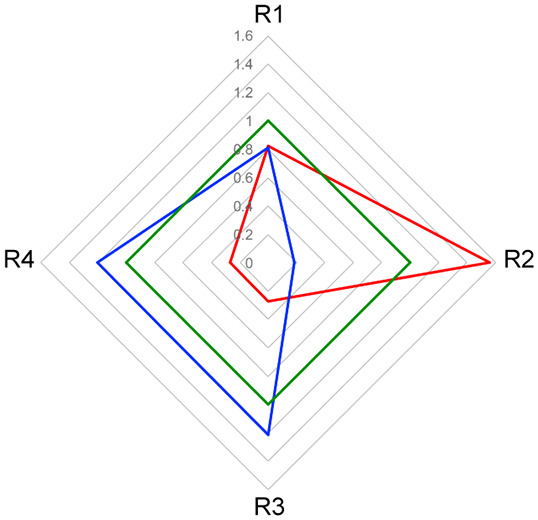

## Introduction

G protein-coupled receptors (GPCRs) are a superfamily of signaling proteins that regulate multiple physiologic events and are one of the most common targets for therapeutic agonists and antagonists. The receptors for bitter tastants (TAS2Rs) are GPCRs that comprise a group of 25 subtypes,^1^ which were initially thought to be exclusively expressed in the oral cavity, but are now known to be expressed on many cell types throughout the body, with activation resulting in physiologically relevant effects. Some examples of cell types known to express one or more physiologically functional TAS2Rs include airway, vascular, uterine, and gastrointestinal smooth muscle cells, cardiomyocytes, enteroendocrine cells, lymphocytes, renal cells, and multiple neuronal tissues.^2–4^ For human airway smooth muscle (HASM) cells, TAS2R5 activation causes marked relaxation and bronchodilation, and decreased smooth muscle mass, suggesting they may be targets for the treatment of obstructive lung diseases.^4^ TAS2Rs couple to members of the heterotrimeric G_i_-family of G proteins, with the released βγ subunit activating phospholipase C β generating inositol 1,4, 5 trisphosphate (IP3). IP3 binds to its receptor on the endoplasmic reticulum causing the release of [Ca^2+^]_i_ to specialized subcellular domains.^4^ The effects from increased [Ca^2+^]_i_ are cell type specific, involving multiple downstream proteins.^5^

TAS2Rs undergo agonist-promoted desensitization (tolerance) of the G protein response during agonist exposure, which could limit the efficacy of administered agonists.^6,7^ The agonist-bound TAS2R undergoes phosphorylation at intracellular sites by G protein-coupled receptor kinases (GRKs), which promotes the binding of β-arrestins to the receptor, competing for G protein binding and thus attenuating (desensitizing) the response.^7^ β-Arrestins also signal independent of G protein activation by assembling complexes due to their chaperone and scaffolding properties.^8,9^ Indeed, for certain GPCR targets, β-arrestin signaling appears to be the basis of the therapeutic effect rather than the G protein-derived signal.^10^ β-Arrestin also orchestrates receptor internalization, leading to signal compartmentalization and ultimately receptor degradation/recycling.^11^ We considered that different TAS2R agonists might stabilize certain conformations that favor one pathway over another (G protein or β-arrestin), a phenomenon known as selective pathway signaling or biased agonist activity.^12^ On a theoretical basis, bias of G protein or β-arrestin signaling could be in either direction (i.e., toward or away from β-arrestin or G protein), but bidirectional biasing has not been reported for TAS2Rs. The current work tested TAS2R5 agonists that displayed different degrees of agonist-promoted desensitization as the readout for disparities in β-arrestin engagement. We examined the molecular basis for two compounds representing the phenotypic extremes of desensitization. We found one compound that has minimal signaling and another that has enhanced signaling to β-arrestin compared to the reference agonist. Furthermore, the intense desensitization evoked by the latter caused extreme biasing with its enhanced β-arrestin and markedly depressed G protein signaling, representing a two-pathway, and dynamic, form of bias. Taken together we demonstrate that signal biasing in either direction to either pathway is possible for TAS2R5 and show that GPCR biasing can be a dynamic process, switching from unidirectional to bidirectional based on β-arrestin actions, further expanding the possibilities for tailored therapy.

## Materials and Methods

### Cell Lines and Agonists

HEK-293T cells were purchased from Millipore Sigma. The immortalized HASM cells (denoted D9) were obtained from A.J. Halayko, University of Manitoba, Winnipeg, Canada.^13^ Both cell lines tested negative for mycoplasma. Seven TAS2R5 agonists which we have previously identified^14^ were screened for agonist-promoted desensitization of the [Ca^2+^]_i_ response in the D9 HASM cells. The structures of these compounds and their previously determined potencies and efficacies are provided in Table S1. Unless otherwise indicated, studies were carried out at 20× the EC_50_ for each agonist.

### Cell Culture and TAS2R5 Desensitization

The HASM cells and HEK-293T cells were grown in DMEM with 10% bovine serum as described.^15^ Transient transfections of HEK-293T cells using lipofectamine were performed exactly as described.^7^ To screen for extremes in the extent of short-term agonist-promoted desensitization in HASM cells, a “pre-treat, wash, treat-again” protocol was utilized, with [Ca^2+^]_i_ as the measure of TAS2R-G protein coupling.^4^ Briefly, cells in monolayers were plated in 96-well plates at ∼20,000 cells per well and 24 h later loaded with Fluo-4 Direct Calcium Dye in HBSS. Cells were incubated in dye for 30 min at 37 °C followed by 20 min at 25 °C. One-half of the 96-well plate was then pretreated for 10 min with agonist at a concentration of 20× the EC_50_ and the other half was treated with vehicle control. Following the pretreatment, cells were washed three times with HBSS at 4 °C, and then phenol-free DMEM at 37 °C was added and the plate was loaded into the FlexStation 3 (Molecular Devices), drugs were dispensed, and immediate readouts of [Ca^2+^]_i_ were obtained in real time for the next 70 s. Drug dispensing was staggered using the column reading method of the plate reader in order to capture the signals across the plate under the same conditions. Stimulations were recorded for the agonists as well as 2.0 μM bradykinin or 2.0 μM ionomycin, which acted as controls. [Ca^2+^]_i_ signals from TAS2R5 agonists were normalized to the ionomycin response. For T5-1, T5-6, and T5-7, which are the focus of the study, treatments with agonist were followed by complete concentration–response curves in the [Ca^2+^]_i_ assay. Desensitization was defined as the extent of the loss of the peak normalized TAS2R5 response as a percent of the response to vehicle pretreatment.

### Receptor Phosphorylation, β-Arrestin Recruitment

HEK-293T cells were transfected with FLAG-TAS2R5 using lipofectamine as described.^7^ After 36 h, cells were treated with vehicle (basal phosphorylation status) or the indicated concentrations of TAS2R5 agonists for 10 min at 28 °C. The total cell lysates were incubated overnight at 4 °C with Phos-tag agarose beads,^16^ washed 3 times with RIPA buffer, and the immunoprecipitate was released with Laemmli stop buffer. Samples were separated using 12% SDS-PAGE, and Western blots were performed using the FLAG antibody. β-Arrestin recruitment was performed as we have described^17^ using the BRET2 method of Bouvier and colleagues.^18^ The β-Arr1-RlucII or β-Arr2-RlucII and rGFP-CAAX expression constructs were transfected into TAS2R5 stably expressing HEK-293T cells using 2 μg of each construct. Cells were incubated in 96-well plates with substrate (Prolume purple) and exposed to vehicle or the indicated concentrations of T5-1, T5-7, or T5-6 for 30 min at 37 °C. BRET data were obtained by signal acquisition at 420 nm (Rluc; donor) and 515 nm (rGFP; acceptor) using a FlexStation 3 (Molecular Devices), and the ratio of the magnitude of the rGFP to Rluc signals was taken as the measure of β-arrestin association. As a control, the human TAS2R5 was mutated to replace all potential phosphoacceptor sites in the third intracellular loop (ICL3) and C-terminal tail (CT) to Ala at positions Ser206, Thr219, Thr282, and Thr290 using previously described methods.^7^

### TAS2R5 Internalization

HEK-293T cells were transfected with a TAS2R5 construct tagged with the 11 amino acid HiBiT peptide at the receptor’s extracellular amino-terminus, which when complemented by LgBiT (derived from luciferase; Promega) added to the cells in culture produces a luminescence signal. Since LgBiT is cell membrane impermeable, the loss of cell surface TAS2R5 from agonist exposure in live cells can be quantitated.^19^ Cells were treated with vehicle or the indicated concentrations of TAS2R5 agonists for 30 min at 37 °C, LgBiT was added to the media, and luminescence was determined. Data are presented as the percent of the signal compared to vehicle. The extreme internalization phenotypes of T5-1 and T5-6 were confirmed by confocal imaging. HEK-293T cells stably transfected with FLAG-tagged TAS2R5 were split onto coverslips and seeded at 25% confluency. Cells were incubated overnight at 5% CO_2_ and 37 °C and the following day treated with agonist (at 20× the EC_50_ concentration) or vehicle for 2 h. The remaining steps were all conducted at 25 °C. Each coverslip was washed 3× with PBS, then fixed with 4% paraformaldehyde and permeabilized with 0.5% NP-40. 10% normal goat serum was applied for 30 min, then the primary antibodies were incubated for 2 h at the following dilutions: FLAG 1:75 (green signal), EEA1 1:50 (red signal). Coverslips were washed 3× in PBS, then secondary antibodies were diluted 1:400 and incubated for 45 min. Cells were washed in PBS and TBS, stained with DAPI (1 μg/mL) for 1 min, and washed with PBS, TBS, and ultrapure H_2_O. Coverslips were then mounted onto slides and imaged using a Leica SP8 Confocal Microscope at 80× magnification. ImageJ software was used to split color channels and the colocalization area was defined as the pixel quantification of red and green overlap at a threshold set to hues 29–43, visually designated as a white signal. Total cell area per image was defined as pixel quantification with no threshold set. Percent colocalization was calculated for each image. Data are from 4 experiments totaling 19–21 images per condition.

### Statistical Analysis

For [Ca^2+^]_i_ assays the peak response over the 70 s of data acquisition for each concentration was determined using Prism10 (GraphPad Software). Images from Western blots and confocal imaging were quantitated using ImageJ software. Concentration–response curves were fit by nonlinear regression using Prism10. When a curve did not plateau, the signal at the highest concentration of agonist was taken as the maximal response. Results were analyzed for statistical significance using ANOVA followed by Tukey’s multiple comparison tests with the adjusted *P* value of <0.05 considered significant. Data are presented as mean ± SEM.

## Results

### Agonist Structure Affects the Extent of Homologous Desensitization of TAS2R5

We have previously shown that the TAS2R14 subtype undergoes agonist-mediated phosphorylation by GRKs at Ser/Thr in the ICL3 and the CT, which initiates desensitization events.^7^ Given the homology between the TAS2R14 and TAS2R5 subtypes, we assumed that this was also in play for TAS2R5 as well. To confirm this, WT and a mutated TAS2R5 (termed 4A, Figure S1A) with Ala substitutions at all four Ser/Thr in the ICL3 and the CT were studied in transfected HEK-293T cells. Agonist-promoted receptor phosphorylation was observed for WT but not for the 4A mutant (Figure S1B,C). Furthermore, pretreatment of cells expressing WT TAS2R5 with inhibitors of GRK2 blocked agonist-promoted phosphorylation, while an inhibitor of GRK5 had a minimal effect (Figure S2A–D). These results suggest that with agonist exposure TAS2R5 undergoes GRK2-mediated phosphorylation events similar to TAS2R14 as well as multiple other GPCRs in the superfamily.^11^ We then proceeded to determine the extent of short-term homologous desensitization of TAS2R5 with a panel of 7 apparent full agonists that we have previously identified.^14^ See Table S1 for structures, potencies, and efficacies of these agonists. For this screen, immortalized HASM cells were pretreated with a TAS2R5 agonist (at 20× the EC_50_) or vehicle for 10 min, washed, and then challenged with the same agonist with an immediate real-time measurement of [Ca^2+^]_i_ performed in the automated plate reader. Two additional second challenges were incorporated in different wells after washing: exposure to bradykinin, which activates [Ca^2+^]_i_ via its receptor, or, the ionophore ionomycin. The former is useful to ensure that the effects observed from TAS2R5 agonist pretreatment are specific to that receptor (i.e., homologous desensitization) and to monitor for potential cell toxicity of the TAS2R5 agonists, while the latter response assesses toxicity, plating density, and other potential nonreceptor/nonspecific events. Figure 1 and Table 1 show the results from these experiments.

**Figure 1:**
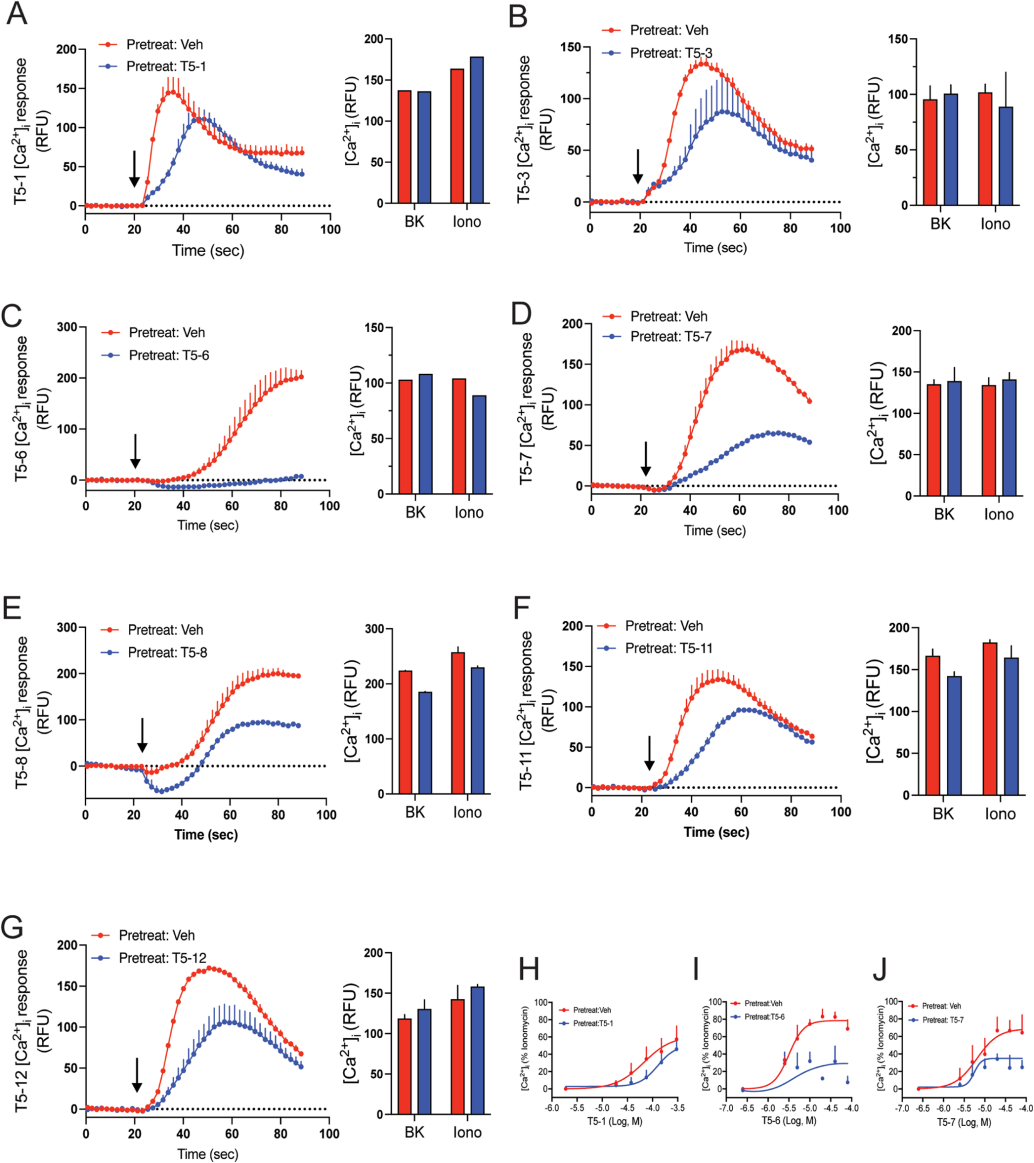
Agonist-promoted desensitization of TAS2R5 by various agonists. A–G) Representative [Ca^2+^]_i_ responses in HASM cells pretreated with vehicle (red data) or the indicated agonists at a concentration of 20× the EC_50_ (blue data) for 10 min. See Table S1 for agonist structures. Cells were then washed and exposed to the same agonist at the same concentration, as well as bradykinin or ionomycin as controls (bar graphs), and [Ca^2+^]_i_ was measured in real time. Data are mean ± SEM of 4 replicates from a representative experiment. See Table 1 for results from multiple experiments. H, I, J) Concetration-response curves from cells challenged with vehicle or the agonists T5-1, T5-6, and T5-7 as above. The results were consistent with the single-concentration studies showing a desensitization rank of T5-6 > T5-7 > T5-1 based upon efficacy. The EC_50_ in the desensitized state increased by 3-fold or less for all three agonists. Results are from 4 experiments.

**Table 1:**
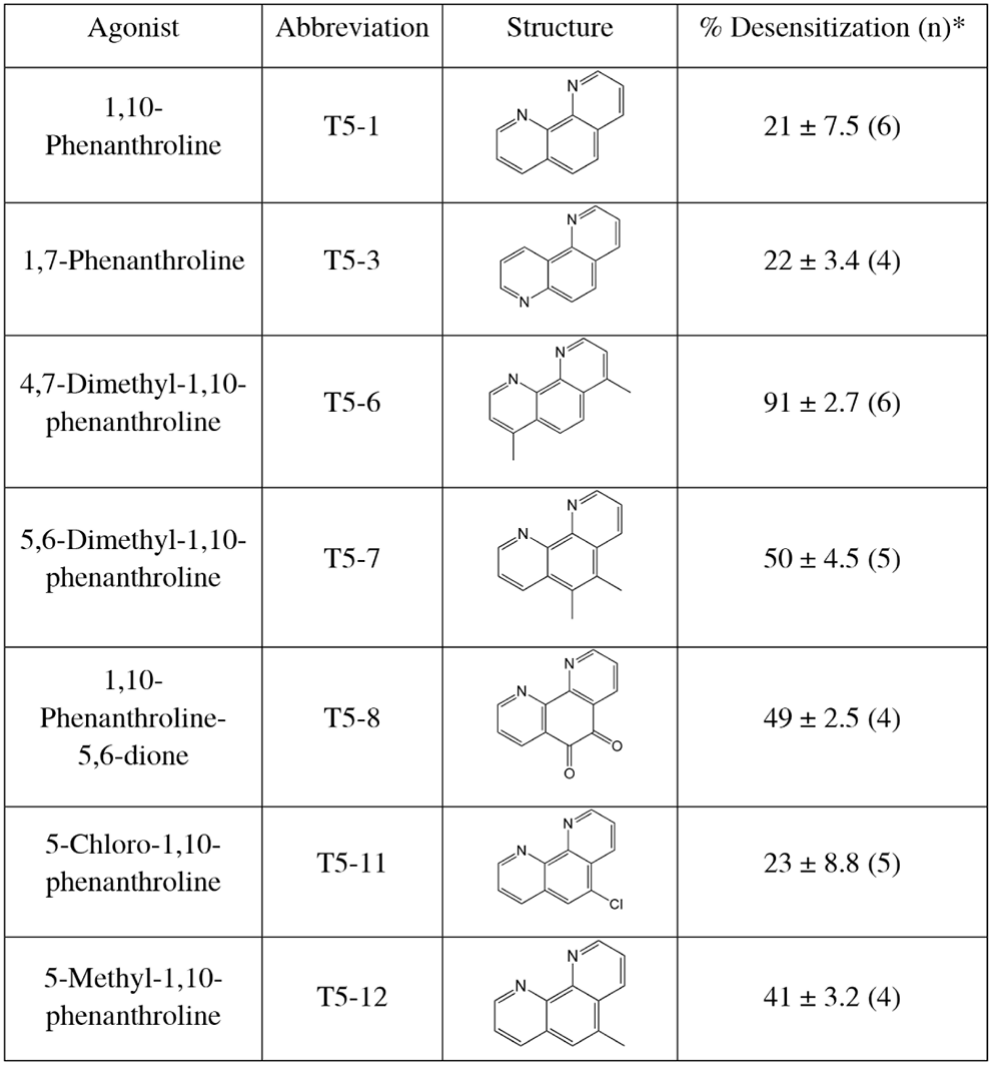
Agonist Structures and Desensitization Responses of HASM Cells to the Indicated Agonists^a^

a The extreme differences in desensitization led us to choose T5-1 and T5-6 (as well as T5-7 for the reference agonist), for further studies. The extents of desensitization were statistically different between these three agonists (*P* < 0.01).

We noted that 1,10-phenanthroline (T5-1) had one of the lowest extents of desensitization amounting to 21 ± 7.5% (Figure 1A and Table 1). At the other extreme, 4,7-dimethyl-1,10-phenanthroline (T5-6) exhibited marked desensitization of 91 ± 2.7% (Figure 1C and Table 1). The other agonists were either similar to T5-1 in the extent of desensitization or had intermediate levels. Minimal to no changes were observed in the bradykinin or ionomycin responses after TAS2R5 agonist treatment (see bar graphs in Figure 1), indicating conditions that identify homologous desensitization of TAS2R5. The agonist designated T5-7 displayed 50 ± 4.5% desensitization and was considered a potential balanced (unbiased) agonist, henceforth termed the reference agonist, for comparing results from other experiments with those from T5-1 and T5-6. Full concentration–response experiments with these three agonists after vehicle or agonist challenge showed similar decreases in the maximal response (see Figure 1H,I,J and legend) confirming the single-concentration responses (Table 1) and a rank order of efficacy for desensitization of T5-6 > T5-7 > T5-1.

### β-Arrestin Recruitment and Internalization are Enhanced with T5-6 and Depressed with T5-1

We measured β-arrestin1 and -2 recruitment by TAS2R5 agonists using a BRET2 assay as described.^18^ HEK-293T cells stably expressing TAS2R5 were transiently transfected to express rGFP-CAAX and β-Arr1-RlucII or β-Arr2-RlucII. Cells were treated with multiple concentrations of agonist for 30 min. Both β-arrestin1 and β-arrestin2 were recruited by the reference agonist T5-7 (Figure 2A,B). In contrast, T5-1 displayed little recruitment over baseline. T5-6 evoked recruitment of both β-arrestins that was significantly greater than the reference (and T5-1). Taken together, the pattern of the maximal β-arrestin recruitment (T5-6 > T5-7 > T5-1) was the same for for recruitment and receptor desensitization. Given that β-arrestin recruitment also initiates receptor internalization, we expected the greatest extent of internalization would be found with T5-6. We measured this property in two ways. TAS2R5 was tagged with the HiBiT peptide at the receptor extracellular amino-terminus, which when complemented by extracellular LgBiT (derived from luciferase) produces a luminescence signal representing cell surface receptors. Cells were exposed to multiple concentrations of the agonists for 30 min. T5-6 evoked a maximum ∼60% loss of cell surface receptors, while T5-1 showed very little (∼10%) reduction (Figure 2C). T5-7 elicited a ∼40% internalization response. T5-6 also had increased potency for internalization compared to T5-7 (EC_50_ = 50 ± 11 μM vs 819 ± 105 μM, respectively) despite similar potencies for G protein-mediated stimulation of [Ca^2+^]_i_ (Table S1), further suggesting a divergence in signaling between the two pathways with this agonist. The significant differences in internalization by T5-1 and T5-6 were confirmed by confocal microscopy (Figure S3), where colocalization of TAS2R5 and the early endosomal marker EEA1 was quantitated after 2 h of agonist exposure. Colocalization was not readily detected with T5-1 exposure (*P* > 0.05 vs vehicle), while colocalization amounting to ∼2-fold over vehicle was readily observed with T5-6 (Figure S3).

**Figure 2:**
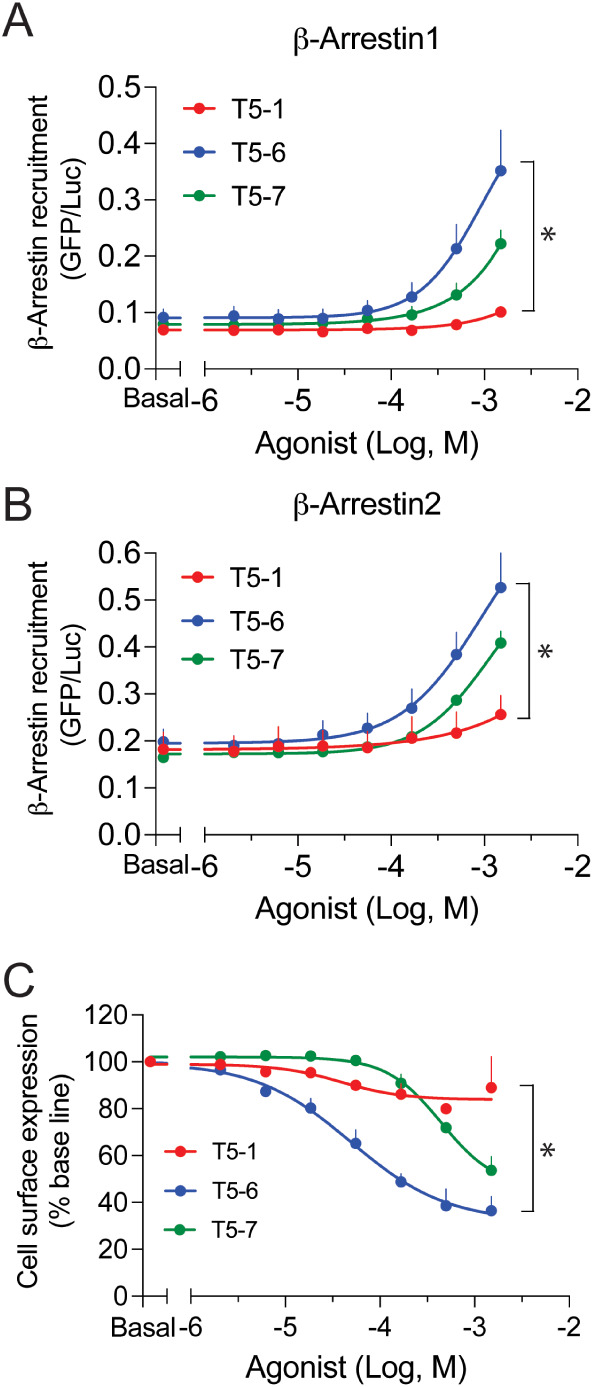
TAS2R5 agonist-promoted β-arrestin1,2 recruitment and internalization differ by TAS2R5 agonist. A,B) HEK-293T cells stably expressing TAS2R5 were transfected with rGFP-CAAX and β-Arr1-RlucII or β-Arr2-RlucII and then exposed to vehicle (basal) or the indicated concentrations of agonist for 30 min at 37 °C in culture. The BRET ratio was calculated as per Methods. The maximal responses (*, *P* < 0.01) differed between each agonist for both β-arrestins. Data are mean ± SEM of 4 experiments. C) HiBiT-TAS2R5 transfected HEK-293T cells were exposed to vehicle or the indicated concentrations of agonists for 30 min at 37 °C. HiBiT-tagged (amino-terminus) TAS2R5 expression on the cell surface was quantified by an enzyme complementation assay, where addition of LgBiT after agonist treatment forms active luciferase. Since LgBiT is membrane impermeable, the loss of luminescence represents internalization. The maximal responses (*, *P* < 0.01) differed between each agonist. Data are mean ± SEM of 3–4 experiments.

### Differential TAS2R5 Desensitization by T5-1 vs T5-6 Is Not due to Variation in Receptor Phosphorylation

For TAS2Rs, GRK phosphorylation as well as a specific conformational change in the third intracellular loop and cytoplasmic tail leads to β-arrestin recruitment to the receptor.^7^ We ascertained agonist-promoted (10 min) TAS2R5 phosphorylation in transfected HEK-293T cells by performing coimmunoprecipitation assays using Phos-tag agarose beads and the FLAG antibody (see Methods). In these experiments two TAS2R5-specific phosphorylated species were observed, potentially representing the monomer and a dimer. All three agonists promoted TAS2R5 phosphorylation at both molecular weights (Figure 3A), and there was no statistical difference in the extent of phosphorylation between T5-1, T5-6, and T5-7 (Figure 3B).

**Figure 3:**
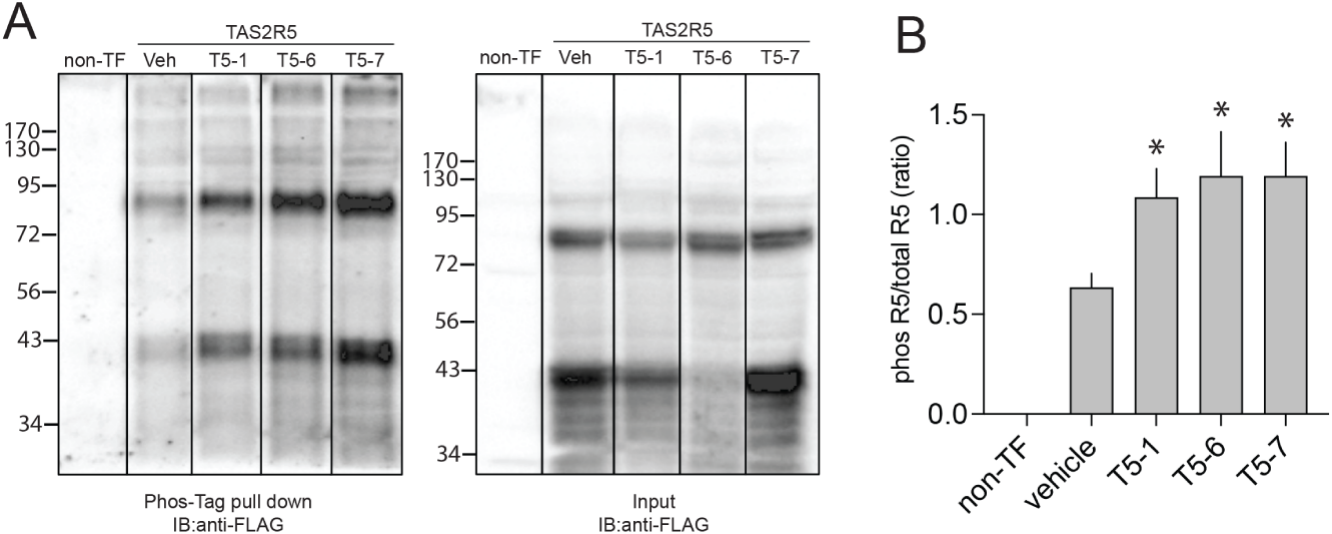
Agonist-promoted TAS2R5 phosphorylation is equivalent between T5-1 and T5-6. FLAG-TAS2R5 transfected HEK-293T cells were treated with vehicle (basal phosphorylation status) or the 20× EC_50_ concentrations of T5-1, T5-6, and T5-7 for 10 min at 28 °C. The total cell lysate was incubated overnight at 4 °C with Phos-tag agarose beads, washed, and the immunoprecipitate released with Laemmli stop buffer. Samples were separated using 12% SDS-PAGE, and Western blots were performed using the FLAG antibody. The three agonists evoked equivalent degrees of phosphorylation of TAS2R5. A) A representative blot from one experiment. B) Results from multiple experiments plotted as the ratio of the phosphorylated TAS2R5 to the TAS2R5 total input. *, *P* < 0.05 vs vehicle, but not different from each other. Shown are mean ± SEM of 3–4 experiments.

### The Dynamic Nature of TAS2R5 Agonist Biasing

Figure 4A shows the results from the indicated assays (maximal responses) and the two agonists plotted against the reference agonist T5-7. The top portion represents G protein signaling in the absence of agonist pretreatment (control). This indicates similar maximal signaling for the agonists T5-1, -6, and -7 under nondesensitizing conditions. The left portion of the figure shows the enhanced and depressed β-arrestin signaling for T5-6 and T5-1, respectively, compared to T5-7. Similarly, internalization follows the same pattern (bottom portion). Without knowledge of the extent of desensitization of the G protein signaling, one would conclude that in terms of the β-arrestin responses, T5-6 is greater than, and T5-1 is less than, the T5-7 reference, while G protein signaling is unaffected by the differences in agonist structures. This represents biasing differences due to variation in one pathway. The extensive homologous desensitization evoked by T5-6 (from increased β-arrestin actions) results in marked depression of the G protein signaling, and in this dynamic scenario, T5-6 exhibits enhanced β-arrestin and decreased G protein signaling, a two-pathway-based bias.

**Figure 4:**
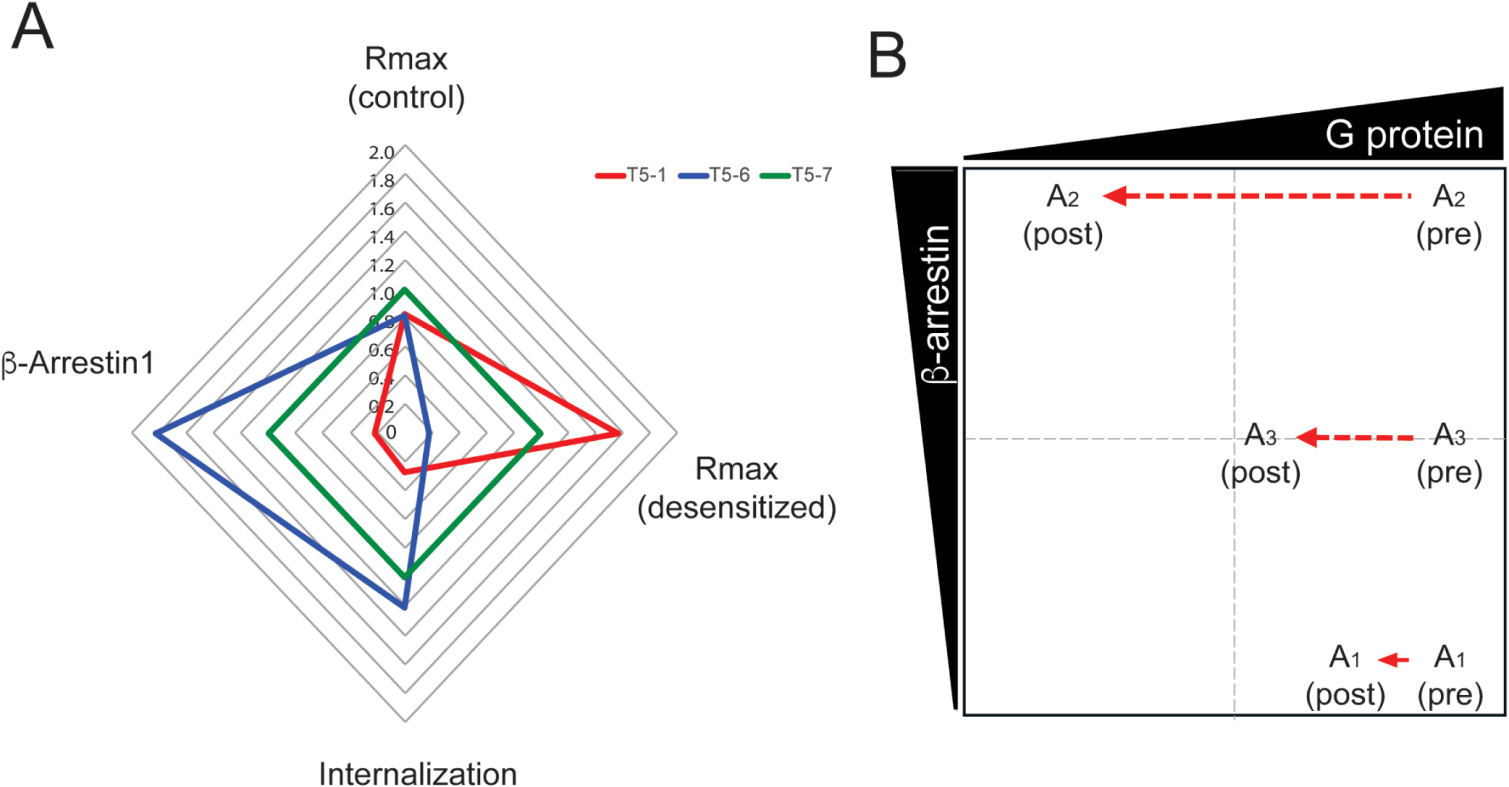
Biasing of TAS2R5 agonists. A) Efficacy radar plot of the four indicated maximal responses for T5-1 and T5-6 normalized to the reference agonist T5-7. In the absence of desensitization, T5-1 and T5-6 have similar coupling to G protein and are biased toward (T5-6) or away (T5-1) from β-arrestin. With desensitization, TAS2R5 G protein coupling from T5-6 is significantly decreased representing a two-pathway effect in opposing directions, leading to greater bias (β-arrestin over G protein). B) General model for dynamic biasing. A_1_ and A_2_ are biased agonists favoring G protein (A_1_) or β-arrestin (A_2_). A_3_ is the reference agonist. See text for discussion. “pre” = function prior to continuous agonist exposure; “post” = continuous agonist exposure followed by washing and rechallenge with agonist.

A general paradigm representing this dynamic biasing is shown in Figure 4B. Here, four possibilities of signaling to two events by the agonists A_1_ and A_2_ are illustrated along with the reference control (A_3_). In the static model, which does not account for the functional desensitization component, A_2_ (such as T5-6) differs from A_1_ (such as T5-1) by the extent of β-arrestin signaling, with equivalent G protein coupling. This represents a one-pathway opposing direction bias, defined only by the difference in β-arrestin signaling (A_1_ decreased and A_2_ increased). However, in the dynamic model, G protein coupling is markedly decreased with A_2_ after agonist-promoted desensitization, transitioning its position to the indicated quadrant representing two-pathway effects in contrasting directions (increased β-arrestin and decreased G protein coupling). Agonist A_3_, the reference agonist, exhibits a typical extent of GPCR desensitization and β-arrestin signaling. For illustrative purposes, A_3_ remains in its predesensitization quadrant although G protein signaling is modestly reduced.

## Discussion

Most GPCRs are now recognized to signal to multiple pathways.^9,20^ For some receptors, the pathways can be selectively activated by structurally distinct agonists, which stabilize the receptor in conformation(s) that favor one or more pathways as compared to others. These biased agonists have the hypothetical capacity to maximize pathways leading toward therapeutic benefit, while potentially avoiding pathways that are detrimental to the disease process, cause adverse side effects, or promote tolerance. Studies with β-arrestin2 KO mice have shown increased analgesia, decreased tolerance or decreased respiratory depression to the μ-opioid receptor (MOR) agonist morphine,^21,22^ with the latter effect in some dispute,^23,24^ potentially due to different study designs. Using WT mice, the MOR agonist SR-17018 was found to be biased away from β-arrestin2 and displays antinociception equivalent to morphine but with less respiratory depression, altered mental status, and tolerance.^25,26^ Similarly, the MOR agonist denoted PZM21 shows little β-arrestin recruitment compared to morphine but efficiently couples to Gαi. Respiratory depression in mice by this agent has been reported to be minimal in some,^27^ but not all studies.^28^ Pathway selectivity is not confined to synthetic agonists, as indicated by studies of various chemokine receptors. The CCR10 receptor, when activated by the endogenous chemokine CXCL28, is biased toward G protein, while the endogenous agonist CCL10 is balanced.^29^ Given the widespread expression of the 25 human TAS2Rs, they are being considered as novel drug targets.^3,30,31^ Yet, we know little about whether agonists acting at these Class T receptors can be devised to selectively activate/deactivate these pathways.

One set of pathways that has been extensively explored with other receptors involves receptor-mediated coupling to G protein and to β-arrestin actions.^9,32^ The latter typically serves at least two functions when activated: 1) desensitizing receptor function by competeing with receptor:G protein interactions, and 2) initiating receptor-dependent, G protein-independent, intracellular events such as receptor internalization. In the current work, we sought to see if TAS2R5 could be biased away from either β-arrestin or from G protein signaling by different agonists. Finding a set of agonists that could achieve either bias would suggest that the TAS2R5 subtype might be a particularly attractive drug target since the receptor is amenable to agonist selectivity regardless of the preferred pathway. In this context, it is unusual to find two full agonists at a single GPCR that can bias signaling in either direction. Agonists chosen for the more extensive studies in this work were obtained by screening a panel of compounds for their efficacy for G protein signaling to [Ca^2+^]_i_ stimulation and their potential to evoke short-term homologous desensitization. We found that the extent of desensitization varied significantly by compound structure. Two agonists with similar efficacies for G protein coupling (defined as having equal maximal stimulation of [Ca^2+^]_i_), T5-1 and T5-6, displayed ∼21% and ∼91% desensitization. T5-7 showed modest (∼50%) desensitization and was considered the reference agonist. We sought to explore the mechanisms of this marked difference in desensitization between T5-1 and T5-6, which we hypothesized was due to different extents of β-arrestin recruitment by the two agonists. The conformation of the receptor necessary for β-arrestin binding is dependent on receptor phosphorylation of intracellular regions by GRKs and the “transmitted” structural information to these intracellular regions imposed by the agonist in the binding pocket and surrounding residues. Due to homology with the TAS2R14 subtype, where we have shown that the GRK sites are Ser/Thr in the third intracellular loop and cytoplasmic tail,^7^ we assumed that this paradigm was active at TAS2R5 as well. Indeed, a mutant TAS2R5 lacking these Ser/Thr failed to undergo agonist-promoted phosphorylation (Figure S1), and WT phosphorylation over baseline was blocked by a GRK2 inhibitor (Figure S2). We found equal receptor phosphorylation induced by T5-1, -6, and -7, yet different extents of β-arrestin1 and β-arrestin2 recruitment and internalization by these agonists. It is possible that different Ser/Thr residues are being phosphorylated by GRKs promoted by the two agonists while total phosphorylation is the same.^33^ On the other hand, it is conceivable that each agonist stabilizes a different conformation of the receptor that ultimately results in different degrees of β-arrestin binding in the face of similar GRK phosphorylation. Regardless of the specific amino acids that are phosphorylated, it is clear that β-arrestin recruitment and internalization are depressed with T5-1 and enhanced with T5-6, consistent with the desensitization profile. TAS2R biasing away from β-arrestin would be advantageous for agonists whose effects are mediated by G protein activation and where desensitization (clinically manifested as tachyphylaxis) would be undesirable. This would be the case for HASM cell relaxation responses in the treatment or prevention of bronchospasm in asthma.^4,6^ β-arrestin cellular actions appear to be context dependent, but have been shown to affect death and proliferation in some cell types.^34^ So, biasing in favor of β-arrestin activation could have a therapeutic advantage, and in fact some TAS2R agonists promote antitumor phenotypes in cancer cells.^35,36^

GPCR agonist biasing is defined as receptor activation of a certain pathway over another pathway, compared to activations from the unbiased reference agonist.^12^ In most reports, this involves the enhancement (or attenuation) of one pathway signal with no change in the other signal. While this scenario does represent biasing that might improve the therapeutic response, it may not decrease adverse effects that are associated with the other signal because it has not changed. A potentially superior biased agonist would evoke a change in one pathway signal and a directionally opposite change in the other pathway signal. The comparison of the two pathways (the bias) would be further enhanced under these circumstances and the therapeutic effectiveness of such an agonist might be improved by this shift in both pathways in contrasting directions. The marked desensitization (∼90%) promoted by T5-6 uncovered this form of biasing for TAS2R5. As indicated in Figure 4A, G protein signaling of T5-6 observed under nondesensitizing conditions was similar to T5-1 and T5-7, and might lead to the conclusion that T5-6 is biased toward β-arrestin while retaining G protein signaling (single-pathway biasing). However, when the dynamic events (over 10 min) leading to receptor desensitization occur, T5-6 has significantly diminished G protein signaling and thus displays the two-pathway effect (increased β-arrestin and decreased G protein signaling). In the dynamic depiction of Figure 4B, the decrease in G protein coupling from desensitization shifts the placement of agonist A_2_ to the indicated quadrant, such that it is biased toward β-arrestin and away from G protein signaling. The agonist A_1_ undergoes little desensitization, so its characterization as a one-pathway biased agonist remains the same. The reference agonist A_3_ is a full agonist for G protein signaling, but promotes a more moderate β-arrestin signal and subsequent desensitization effect, so it remains in the same quadrant. Depending upon where the transition boundaries are set, though, A_3_ could shift. Indeed, this dynamic aspect became apparent to us primarily because of the rapid and extensive desensitization evoked by T5-6. This brings up the proposition that agonists which might be considered unbiased become biased over time if they evoke sufficient desensitization from any mechanism. Our findings do not appear to be confined to TAS2Rs. A temporal component has also been described for various signals evoked by agonists acting at the D_2_ dopamine receptor,^37^ although these were not tied to homologous receptor desensitization per se. Nevertheless, the collective findings with two structurally dissimilar receptors from class A and class T suggest that the phenomenon may hold across the superfamily.

## Conclusions

We have shown that TAS2R5 signals to G protein and β-arrestin. The extent of this signaling is significantly affected by agonist structure compared to a reference agonist, with one agonist biasing toward and another agonist biasing away from β-arrestin with initially intact G protein coupling. Over time, the extensive β-arrestin signal feeds back to desensitize receptor coupling to the G protein, which markedly biases the receptor from two-pathway events. This demonstrates an additional complexity to biasing, showing that the two pathways can be selectively affected in contrasting directions by a single agonist. We term this dynamic biasing to indicate how the biased characteristics of drugs can change over time, which may ultimately impact their clinical utility.

## Supplementary Material



## Notes

Table S1 structures and function of the seven agonists; Figure S1 phosphorylation of TAS2R and the 4A mutant; Figure S2 effects of GRK inhibitors on TAS2R phosphorylation; Figure S3 confocal microscopy of TAS2R and EE1 colocalization (PDF)
