# Supplementary material for: Dynamic Pathway Selectivity of TAS2R5 toward or Away from β‑Arrestin or G Protein from Biased Agonists
Source: Biochemistry. 2026 May 16;65(11):1778–86. doi: 10.1021/acs.biochem.6c00174 (PMC13235537; doi:10.1021/acs.biochem.6c00174)
Supplement: Supplementary file 1 [file bi6c00174_si_001.pdf]

Supporting Information

Dynamic Pathway selectivity of TAS2R5 towards or away from β-arrestin or G protein from biased agonists

Donghwa Kim[a,b], *Hannah R. Strzelinski*[a,b], *Camille M Longabardi*[a,b], *Stephen B. Liggett*[a,b,c,*]

[a]Department of Internal Medicine, University of South Florida Morsani College of Medicine, Tampa, FL 33602, USA;

[b]Center for Personalized Medicine and Genomics, University of South Florida Morsani College of Medicine, Tampa, FL 33602, USA;

[c]Department of Molecular Pharmacology and Physiology, University of South Florida Morsani College of Medicine, Tampa, FL 33602, USA;

[*] Corresponding author; sliggett@usf.edu

Table S1.

Abbreviation	Agonist	Structure	EC_{50} (μM)	Rmax (% of Ionomycin)
T5-1	1,10-Phenanthroline	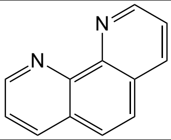	30 ± 5.6	149 ± 10.2
T5-3	1,7-Phenanthroline	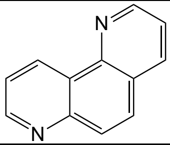	25 ± 4.6	122 ± 16.7
T5-6	4,7-Dimethyl-1,10-phenanthroline	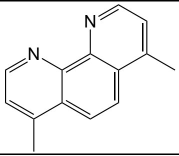	1.4 ± 0.19	163 ± 15.1
T5-7	5,6-Dimethyl-1,10-phenanthroline	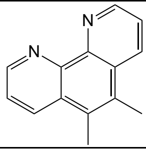	2.01 ± 0.20	163 ± 36.5
T5-8	1,10-Phenanthroline-5,6-dione	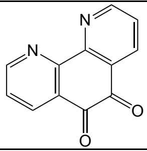	0.12 ± 0.05	200 ± 20.2
T5-11	5-Chloro-1,10-phenanthroline	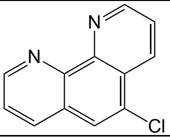	11 ± 2.0	190 ± 13.8
T5-12	5-Methyl-1,10-phenanthroline	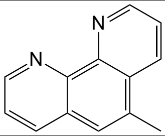	12 ± 2.0	181 ± 24.7

Table S1. Characteristics of the agonists utilized in the current study derived from experiments in HASM cells.

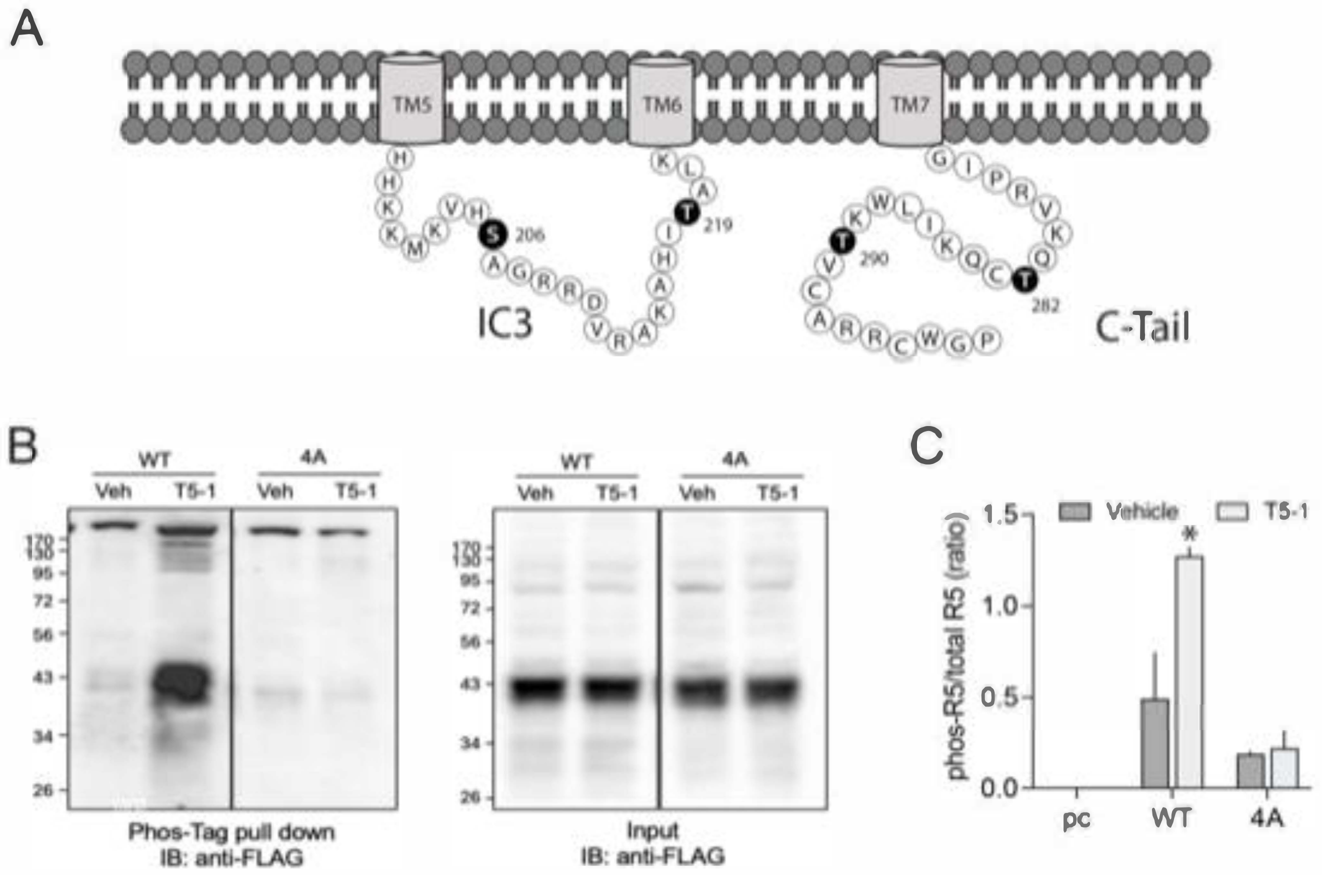

Figure S1. Agonist-promoted phosphorylation of TAS2R5 is dependent upon Ser/Thr in the ICL3 and CT . A) The location of Ser/Thr in ICL3 and CT of TAS2R5 that were substituted with Ala in the TAS2R5-4A mutant. B) Wild-type (WT) FLAG-TAS2R5 exhibits agonist-promoted receptor phosphorylation while the FLAG-TAS2R5-4A mutant does not. Transfected HEK-293T cells were treated with 600 uM T5-1 for 10 min and prepared as described in Methods. Shown are representative western blots of the Phos-Tag immunoprecipitate probed with FLAG antibody (left) and the expression of the receptors prior to the pull-down (right). C) results from 4 experiments. *, P <0.01 for phosphorylation levels greater than baseline (vehicle).

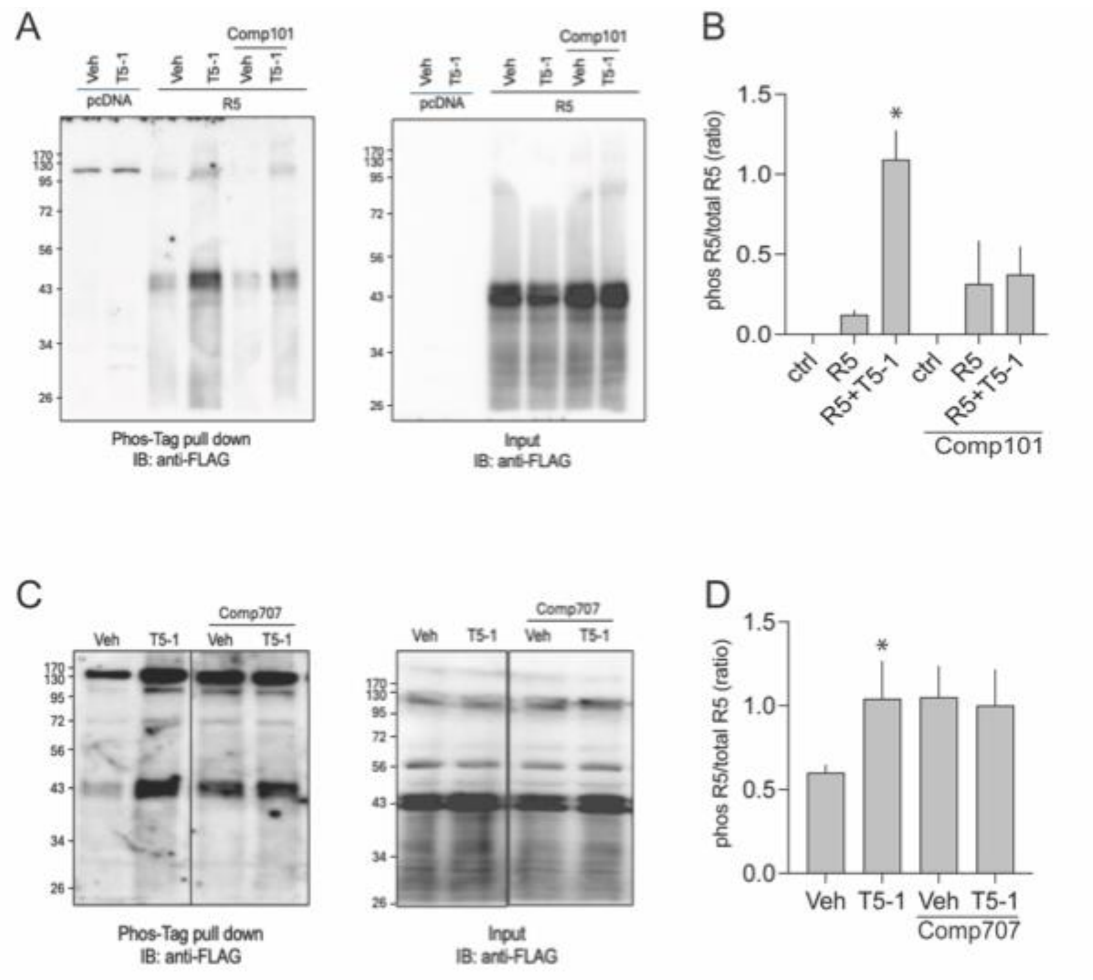

Figure S2. Agonist-promoted phosphorylation of TAS2R5 is attenuated by inhibitors of GRK2 and GRK5. Transfected HEK-293T cells were treated with 600 uM T5-1 for 10 min and prepared as described in Methods. A) Representative western blots from phosphorylation experiments performed in the absence or presence of 10 uM incubation (2 hours) of the GRK2 inhibitor Compound 101. B) Results from 3 experiments. C,D) same as A,B) except with 10 uM of the GRK5 inhibitor Compound 707. *, P <0.01 vs baseline (vehicle). Ctrl, empty vector control.

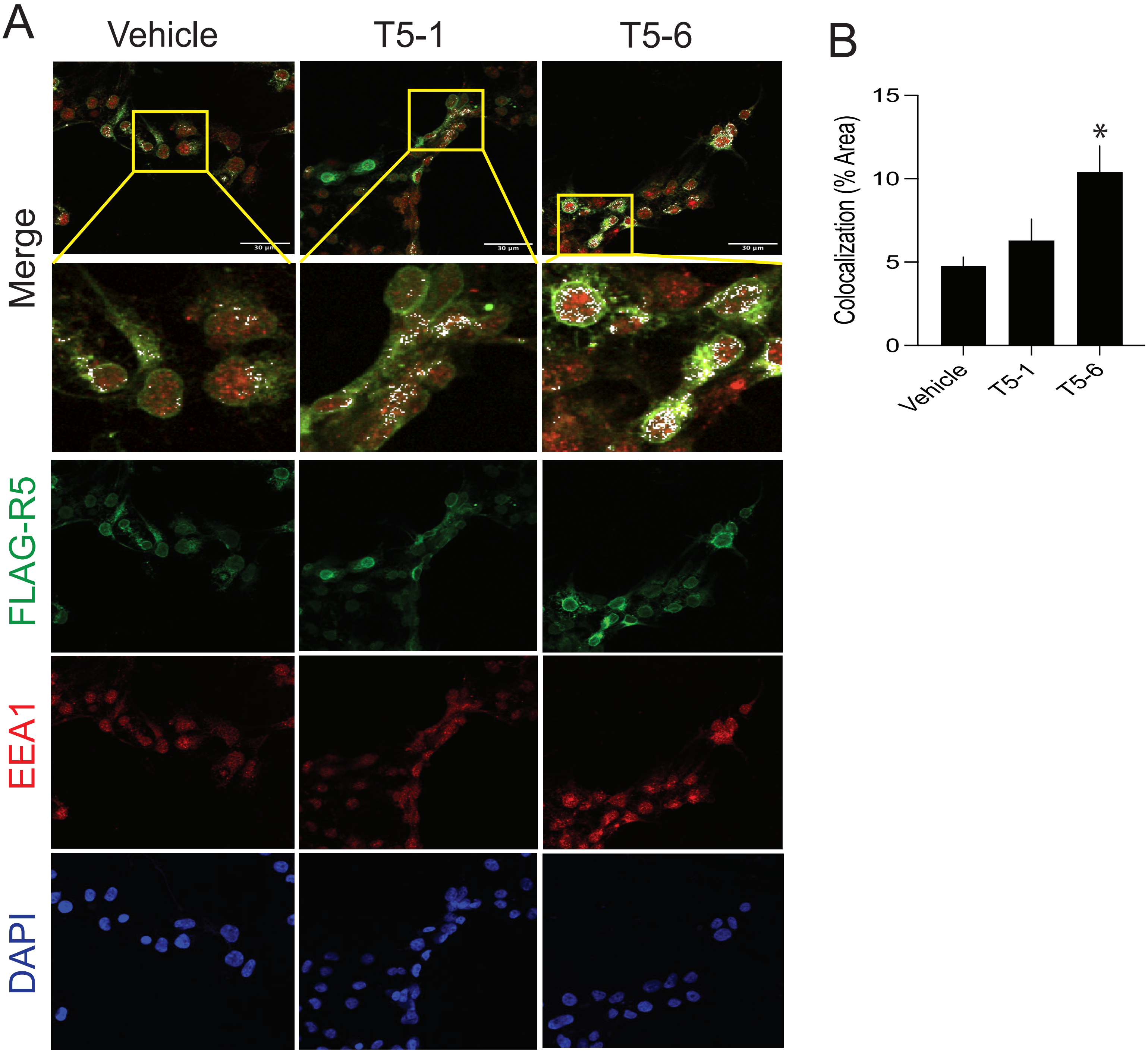

Figure S3. Agonist-promoted colocalization of TAS2R5 with the early endosomal marker EEA1 differs between T5-1 and T5-6. A) Transfected FLAG-TAS2R5 HEK-293T cells were treated with the indicated agonist for 2 hours. Permeabilized cells were probed for FLAG (green signal), EEA1 (red signal), and stained with DAPI and imaged by confocal microscopy. The white signal in the merged images indicates colocalization. Shown are results from a representative experiment. Bar = 30 μm. B) Results from 4 independent experiments consisting of ~100 images. *, P<0.01 vs vehicle
